# Fistula recurrence, pregnancy, and childbirth following successful closure of female genital fistula in Guinea: a longitudinal study

**DOI:** 10.1016/S2214-109X(17)30366-2

**Published:** 2017-09-21

**Authors:** Alexandre Delamou, Therese Delvaux, Alison M El Ayadi, Vandana Tripathi, Bienvenu S Camara, Abdoul H Beavogui, Lauri Romanzi, Bethany Cole, Patrice Bouedouno, Moustapha Diallo, Thierno H Barry, Mandian Camara, Kindy Diallo, Alain Leveque, Wei-Hong Zhang, Vincent De Brouwere

**Affiliations:** Centre National de Formation et de Recherche en Santé Rurale de Maferinyah, Forécariah, Guinea, Ecole de Santé Publique, Université Libre de Bruxelles (ULB), Brussels, Belgium, Maternal & Reproductive Health Unit, Institute of Tropical Medicine, Antwerp, Belgium; Maternal & Reproductive Health Unit, Institute of Tropical Medicine, Antwerp, Belgium; Bixby Center for Global Reproductive Health, University of California, San Francisco, CA, USA; EngenderHealth, New York, NY, USA; Centre National de Formation et de Recherche en Santé Rurale de Maferinyah, Forécariah, Guinea; Centre National de Formation et de Recherche en Santé Rurale de Maferinyah, Forécariah, Guinea; EngenderHealth, New York, NY, USA; EngenderHealth, New York, NY, USA; Centre National de Formation et de Recherche en Santé Rurale de Maferinyah, Forécariah, Guinea; EngenderHealth, Conakry, Guinea; Hôpital Prefectoral de Kissidougou, Kissidougou, Guinea; Centre Medico-Social Jean Paul II, Conakry, Guinea; Hôpital Regional de Labé, Labé, Guinea; Ecole de Santé Publique, Université Libre de Bruxelles (ULB), Brussels, Belgium; Ecole de Santé Publique, Université Libre de Bruxelles (ULB), Brussels, Belgium; Maternal & Reproductive Health Unit, Institute of Tropical Medicine, Antwerp, Belgium

## Abstract

**Background:**

Female genital fistula is a devastating maternal complication of delivery in developing countries. We sought to analyse the incidence and proportion of fistula recurrence, residual urinary incontinence, and pregnancy after successful fistula closure in Guinea, and describe the delivery-associated maternal and child health outcomes.

**Methods:**

We did a longitudinal study in women discharged with a closed fistula from three repair hospitals supported by EngenderHealth in Guinea. We recruited women retrospectively (via medical record review) and prospectively at hospital discharge. We used Kaplan-Meier methods to analyse the cumulative incidence, incidence proportion, and incidence ratio of fistula recurrence, associated outcomes, and pregnancy after successful fistula closure. The primary outcome was recurrence of fistula following discharge from repair hospital in all eligible women who consented to inclusion and could provide follow-up data.

**Findings:**

481 women eligible for analysis were identified retrospectively (from Jan 1, 2012, to Dec 31, 2014; 348 women) or prospectively (Jan 1 to June 20, 2015; 133 women), and followed up until June 30, 2016. Median follow-up was 28·0 months (IQR 14·6–36·6). 73 recurrent fistulas occurred, corresponding to a cumulative incidence of 71 per 1000 person-years (95% CI 56·5–89·3) and an incidence proportion of 18·4% (14·8–22·8). In 447 women who were continent at hospital discharge, we recorded 24 cases of post-repair residual urinary incontinence, equivalent to a cumulative incidence of 23·1 per 1000 person-years (14·0–36·2), and corresponding to 10·3% (5·2–19·6). In 305 women at risk of pregnancy, the cumulative incidence of pregnancy was 106·0 per 1000 person-years, corresponding to 28·4% (22·8–35·0) of these women. Of 50 women who had delivered by the time of follow-up, only nine delivered by elective caesarean section. There were 12 stillbirths, seven delivery-related fistula recurrences, and one maternal death.

**Interpretation:**

Recurrence of female genital fistula and adverse pregnancy-related maternal and child health outcomes were frequent in women after fistula repair in Guinea. Interventions are needed to safeguard the health of women after fistula repair.

**Funding:**

Belgian Development Cooperation (DGD), Institute of Tropical Medicine of Antwerp (ITM), and Maferinyah Training and Research Center in Rural Health (Guinea).

## Introduction

Female genital fistula generally occurs after prolonged obstructed labour, resulting in continuous and uncontrolled leakage of urine or faeces, among other debilitating sequelae.^1,2^ Over the past decade, substantial international mobilisation towards achievement of a fistula­free generation has resulted in improved management of fistula cases,^3^ with high incidence of closure at time of hospital discharge^4–6^ and accomplishment of more than 100 000 surgical fistula repairs in sub­Saharan Africa and south Asia.^7,8^

As more women access fistula treatment worldwide,^9^ attention during the post­repair period is important to ensure health after surgery. Fistula recurrence is of particular interest if the surgical site breaks down or if the woman develops a new, second fistula following mismanaged obstructed labour after previous fistula repair.^10–13^ After successful fistula repair, many women of reproductive age^6^ return to their communities with the hope of resuming their social roles, including conceiving again, possibly to compensate for the traumatic loss they experienced during the delivery that led to the fistula.^10,14–17^

Although there are many data for residual fistulas or failed repairs, few data exist for recurrent fistulas after a successful repair—this paucity might be for various reasons, including varying study designs and case definitions or length of follow­up.^10,11,18,19^ Similarly, data for fertility or pregnancy and childbirth after successful fistula repair are scarce, especially from robust studies that are able to provide a precise estimate of pregnancy and delivery outcomes.^10,14,18,20–22^ A review^23^ in sub­Saharan Africa found that the risk of adverse maternal and neonatal health outcomes was elevated in women after fistula surgery, and that post­surgical fistula recurrence was the most common maternal complication, occurring in 5% of deliveries.^23^ Although the general recommendation to women after fistula repair is to seek care at health­care facilities and deliver via scheduled caesarean section for any post­repair pregnancies,^13,21,22^ the proportion of women delivering via elective caesarean section is low (45%).^23^

It is not known when or under what circumstances recurrence of fistula unrelated to acute, postoperative surgical site breakdown is most likely to occur. Further­ more, data for pregnancy and management of delivery after repair are lacking. Such data are needed to inform holistic fistula prevention and management programmes in countries in which female genital fistula is still prevalent and incident.^24^ Guinea has high maternal mortality (724 maternal deaths per 100 000 livebirths) and a lifetime prevalence of self­reported obstetric fistula symptoms that is double that reported in sub­Saharan Africa as a whole (6·0 [95% CI 3·9–7·4] per 1000 women of reproductive age in Guinea *vs* 3·0 [1·3–5·5] per 1000 women of reproductive age in sub­Saharan Africa).^25,26^ In 2013, the fistula care project implemented by EngenderHealth, funded by the US Agency for International Development, supported three of the four repair hospitals in the country: Jean Paul II Hospital (Conakry), Labé Regional Hospital (Labé), and Kissidougou Prefectural Hospital (Kissidougou). About 3000 fistula repairs were done at the sites between 2007 and 2013.^7^ Additional funding was secured by EngenderHealth to support fistula repairs in 2014–15. Therefore, we did a longitudinal study^19^ among women discharged with a closed female genital fistula from Guinean repair hospitals, with the aim to estimate the incidence of fistula recurrence, residual urinary incontinence, and pregnancy after successful closure of the fistula, estimate the relative contribution of associated factors, and describe delivery­associated maternal and child health outcomes.

## Methods

### Study design and participants

We did a longitudinal observational study among women who underwent fistula repair between Jan 1, 2012, and June 30, 2015, at the three hospitals in Guinea supported by EngenderHealth (Conakry, Guinea). A detailed description of the study setting and methods has been previously published.^19^

We included women with a single genital fistula confirmed to be closed via dye test at the time of discharge from one of the three repair hospitals supported by EngenderHealth, who resided in Guinea.^27^ We excluded women with incomplete medical records, and those who had fistula repair at other sites or who declined consent. Costs for surgery, transportation, and hospital stay for women were fully covered by EngenderHealth. Women were recruited both retrospectively and prospectively. Information on the status of the fistula at discharge was obtained through medical records review (retrospective inclusion) or directly at discharge (prospective inclusion). Ethics approval was obtained from the Institute of Tropical Medicine (ITM) of Antwerp (IRB#948/14), the Ethics Committee of the University Hospital of Antwerp (Ref#14/22/238), and the National Ethics Committee for Health Research of Conakry, Guinea (Ref#10/CNERS/14). Eligible women provided written informed consent.

### Procedures

The study procedures are described in detail elsewhere.^19^ Briefly, the study team contacted eligible women by phone or home visit in their communities across Guinea to obtain informed consent. The study team included nurses involved in the management of women at the fistula repair hospitals, doctors, and final year medical students. According to the protocol, data collection follow­up visits were intended to be done every 6 months. However, because of the ongoing Ebola virus outbreak with its associated community reluctance and resistance, this was not possible. We expected most women to receive one follow­up data collection visit, but depending on timing of participant recruitment some could receive two follow­up data collection visits to maximise length of follow­up. The maximum possible study follow­up was 4·5 years (Jan 1, 2012, to June 30, 2016).^19^

### Outcomes

The primary outcome was recurrence of fistula following discharge from the repair hospital. For this study, recurrence of fistula was defined as the breakdown of a repaired fistula or the occurrence of a new fistula. During follow­up visits, women were first asked about their current continence status with the question, “Do you have continuous and uncontrolled leakage of urine and/or faeces?” If the answer was yes, a dye test for confirmation of fistula (*vs* residual urinary incontinence) was performed at the nearest health­care centre or health post by a member of the research team. The secondary outcomes were time to pregnancy, pregnancy outcome, maternal and neonatal outcomes at first delivery after repair, and residual urinary incontinence among women continent at discharge. Pregnancy was documented by a positive pregnancy test or self­report, and time to pregnancy was calculated from the time of hospital discharge. Residual incontinence was confirmed by a dye test. We also evaluated number of pregnancies per woman, and antenatal care receipt, location of delivery, and method of delivery for each subsequent pregnancy.

Enrolment and follow­up data were collected by trained data collectors by use of structured and pre­tested standardised questionnaires. Sociodemographic data captured at enrolment included age at fistula surgery, level of education, marital status, occupation, and residence (rural or urban). Clinical characteristics at enrolment included number of pregnancies, parity, duration of fistula symptoms, number of previous repairs, mode of delivery during the birth when the fistula occurred, neonatal outcome at this delivery, type of fistula (vesicovaginal fistula, rectovaginal fistula, or both), and continence status at the time of discharge (continent or not continent). The follow­up questionnaire evaluated participants’ current fistula and continence status (fistula closed and continent, closed but not continent, or not closed), self­reported circumstances of fistula recurrence, postoperative and sociodemographic and reproductive characteristics, such as current residence (urban or rural), marital status, occupation, post­repair pregnancies (ongoing, aborted or miscarried, delivered), neonatal outcomes at first delivery post­repair (livebirth, stillbirth, neonatal death), and sex of the child at first delivery post­repair. For individuals who received two follow­up data collection visits, data from the second visit only (to avoid double reporting) was included in the analysis.

### Statistical analysis

We estimated that the minimum sample size required determined by specified precision level (2% margin of error and 95% CI) was 364 women receiving surgical fistula repair.^19^

All women who met eligibility criteria and who were able to be located and interviewed were used in fistula recurrence­related analyses, whereas pregnancy­related analyses were restricted to women of reproductive age who were considered at risk of pregnancy by self­report of sexual activity after repair. We present categorical data as n (%) and compared them with χ^²^ or Fisher’s exact tests. We present continuous data as means with SD (and compared them with Student’s *t* test) and medians with IQR (Mann­Whitney *U* test). p<0·05 was regarded as significant. Among eligible women, we compared sociodemographic and clinical characteristics between women included in our analytical sample and women not included to check for differences at inclusion. We calculated follow­up time from the date of hospital discharge. For calculation of person­time at risk, fistula recurrence, post­repair residual urinary incontinence, or first post­repair pregnancy cumulative incidence and proportion, we considered the self­reported date of onset of recurrent incontinence symptoms or the self­reported first date of last menses as dates of event. Patients who did not experience fistula recurrence or pregnancy, or who died, were censored at the date of last follow­up visit. For all time­related variables, the 15th of the month was used when an exact date was not provided. The study outcomes were estimated as cumulative incidence with Kaplan­Meier survival analysis methods or as proportions. Additionally, we derived incidence ratios of study outcomes and compared them for selected variables using Fisher’s exact test. We carried out an analysis that takes the competing event (one death) into account^28^ but found no difference because of the small number of competing events for fistula recurrence and pregnancy. Study data were managed by EpiData software version 3.1 (EpiData Association, Odense, Denmark) and the analyses were performed using Stata 13 software (Stata Corporation, College Station, TX, USA). This study was registered with ClinicalTrials.gov, number NCT02686957.

### Role of the funding source

The funder of the study had no role in study design, data collection, data analysis, data interpretation, or writing of the report. The corresponding author had full access to all the data in the study and had final responsibility for the decision to submit for publication.

## Results

**Figure 1:**
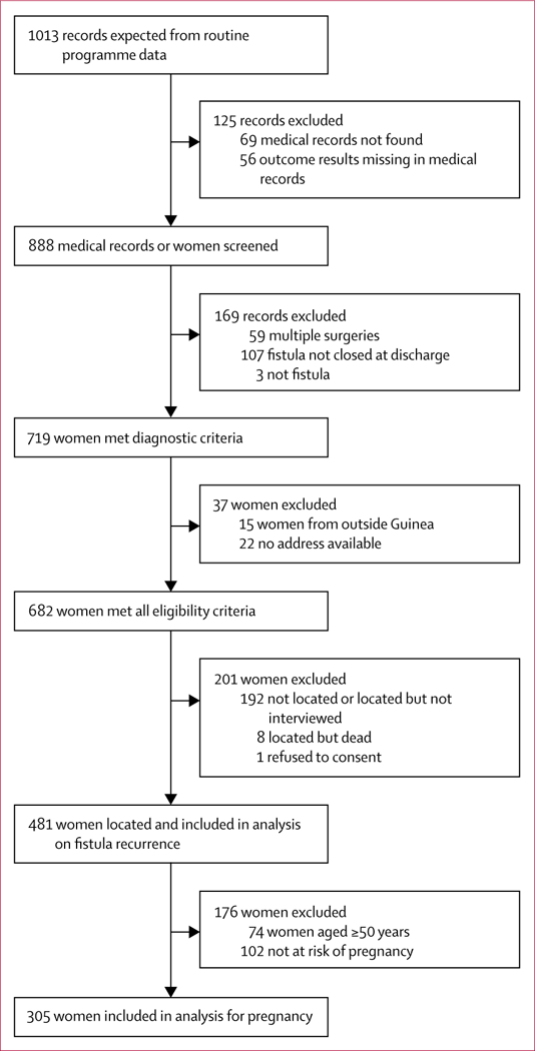
Study profile

Women were recruited both retrospectively (Jan 1, 2012, to Dec 31, 2014) and prospectively (Jan 1 to June 30, 2015), with follow­up ending on June 30, 2016. Overall, the medical records of 888 women were screened (figure 1), of whom 481 (70%) were locatable and consented to inclusion in the analysis for the primary outcome. Of these women, 305 (75%) of reproductive age reported being sexually active after surgery and were considered in the pregnancy­related analyses. Included women came from across the country (appendix). 327 (68%) women received one follow­up visit and 154 (32%) received two.

Table 1 shows sociodemographic and clinical characteristics at time of fistula surgery for eligible women included in the study and eligible women who did not participate. Characteristics were similar in both groups: most women were married or in union, were housewives, and had vesicovaginal fistulas. Most women were continent at hospital discharge, but a small number had residual incontinence. Eligible study participants had experienced more stillbirths during the delivery leading to the fistula than had eligible women not participating in the study.

At follow­up, some sociodemographic characteristics of women included in the study had changed. The proportion of women reporting urban residence had doubled and the percentage of women reporting an occupation other than housewife had increased (table 2).

**Figure 2:**
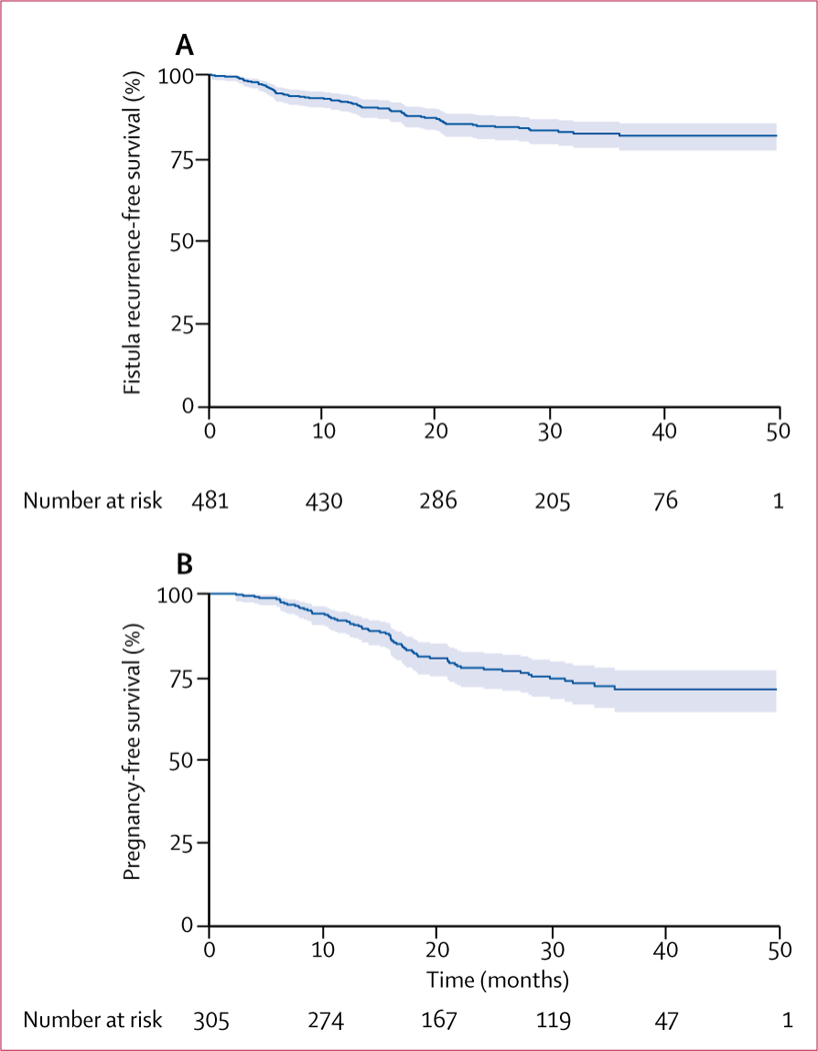
Kaplan-Meier curves for overall recurrence-free survival (A) and first post-repair pregnancy-free survival (B) Shaded regions are 95% CIs.

**Figure 3:**
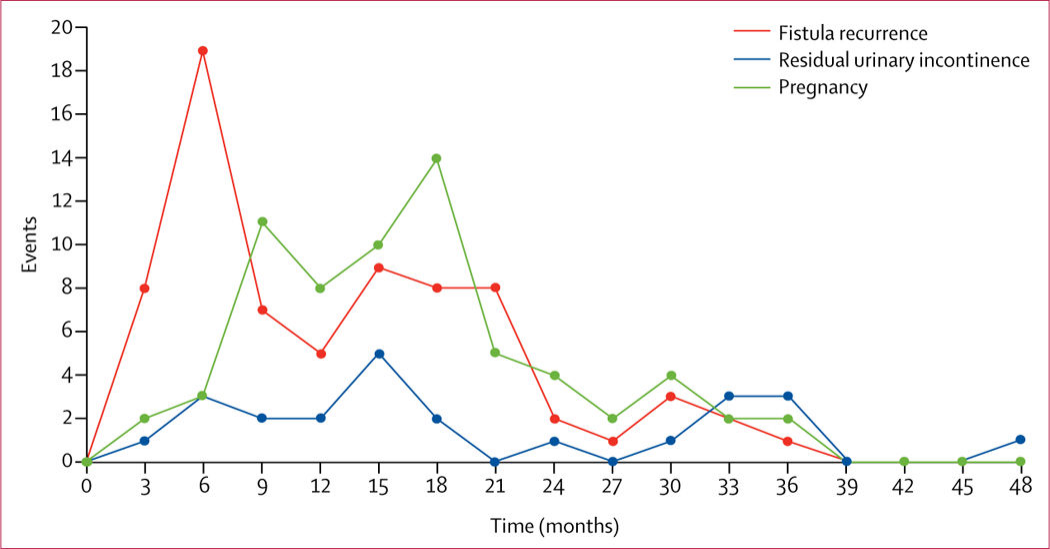
Incidence of fistula recurrence (n=73), first post-repair pregnancy (n=67), and residual urinary incontinence (n=24) over time in study participants

Median follow­up was 28**·**0 months (IQR 14**·**6–36**·**6). The cumulative incidence of fistula recurrence was 71·0 per 1000 person­years (95% CI 56**·**5–89**·**3), corresponding to 18**·**4% (14**·**8–22**·**8) of women (figure 2, table 3). 39 (53%) of the 73 recurrences of fistula occurred during the first 12 months after discharge (27 [37%] during the first 6 months; figure 3). 14 (19%) women self­reported that the recurrence of fistula occurred during farm work, nine (12%) when walking, seven (10%) after sexual intercourse, and seven (10%) after pregnancy and delivery that occurred after the index fistula repair surgery.

We recorded 24 cases of post­repair residual urinary incontinence among 447 women who were continent at hospital discharge, which is equivalent to a cumulative incidence of 23**·**1 per 1000 person­years (95% CI 14**·**0–36**·**2) or 10**·**3% (5**·**2–19**·**6) of women (table 3). Of these 24 cases, eight (33%) occurred during the first 12 months after discharge (figure 3).

Cumulative incidence of residual urinary incontinence did not differ by pregnancy status, sexual activity, urethral involvement, status of the bladder neck, or vaginal scarring (table 4). However, incidence of fistula recurrence was increased in women not sexually active at follow­up, those who had a damaged urethra at fistula surgery, those who had a damaged bladder neck at fistula surgery, and those who had vaginal scarring at fistula surgery (table 4).

**Figure 4:**
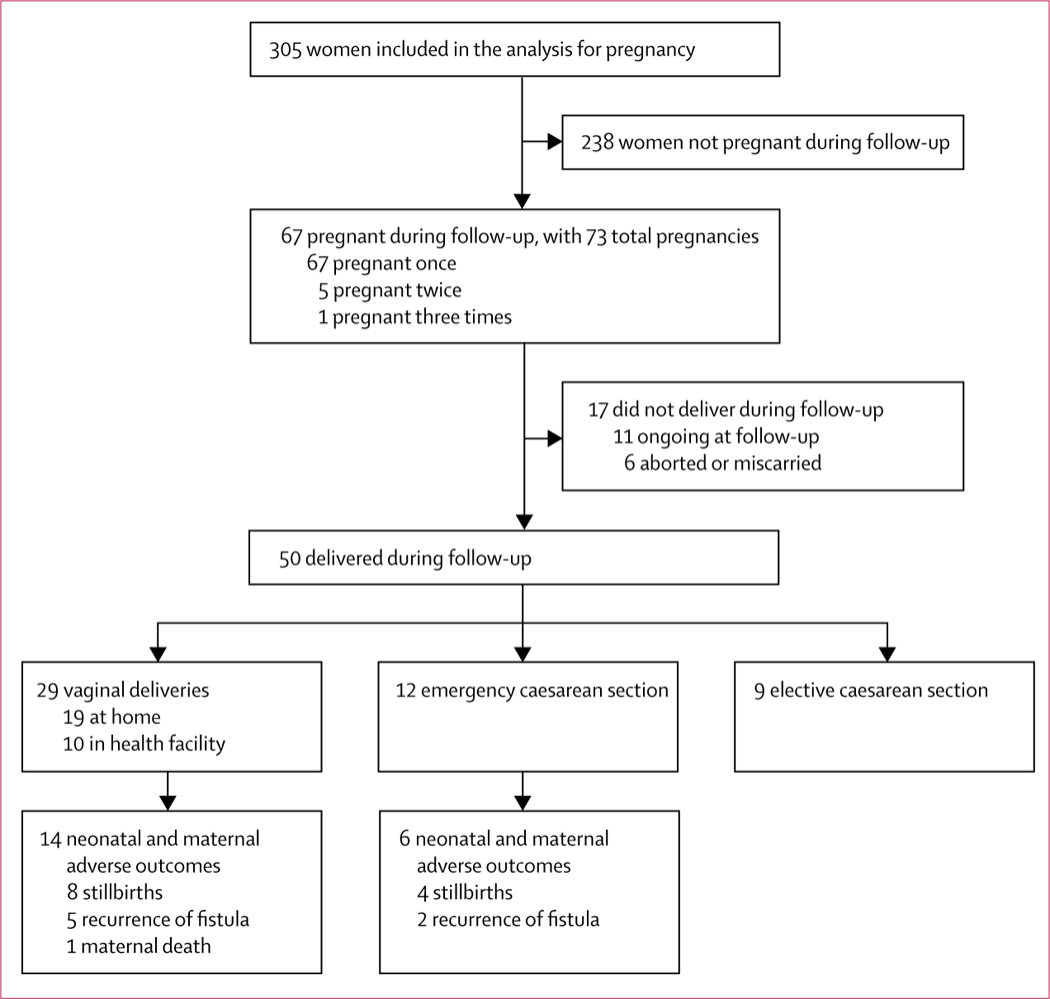
Post-repair pregnancy and delivery outcomes among sexually active study participants of reproductive age

Cumulative incidence of pregnancy was 106**·**0 per 1000 person­years (95% CI 83**·**2–134**·**3), corresponding to 28**·**4% (22**·**8–35**·**0) of women (figure 2, table 3). First post­repair pregnancies occurred between 3 months and 36 months after hospital discharge, with 48 (72%) of the first post­repair pregnancies occurring within the first 18 months, and 57 (85%) within the first 24 months (figure 3). Cumulative incidence of pregnancy did not differ according to urethral involvement, status of the bladder neck, vaginal scarring, or fistula status at the time of hospital discharge (table 4). Of the 67 women with at least one post­repair pregnancy, 51 (76%) achieved at least one antenatal care visit for the first post­repair pregnancy. 50 women had delivered by the time of follow­up, of whom only nine (18%) delivered by elective caesarean section (figure 4). Among these 50 deliveries, we recorded 12 (24%) stillbirths,seven (14%) delivery­related fistula recurrences, and one (2%) maternal death.

## Discussion

This study found that fistula recurrence was quite frequent among women who underwent fistula repair in Guinea, with a higher incidence than expected. Low recurrence rates were expected given the counselling done before surgery and at hospital discharge, and also women’s knowledge of the devastating effects of fistulas. Existing literature does not provide cumulative incidence for fistula recurrence. However, by 24 months’ follow­up, we recorded a cumulative incidence of 15**·**5% (95% CI 12**·**3–19**·**4) of women compared to 3**·**9% reported in a small study^10^ of 26 women followed up for 9–24 months post­repair in Malawi and 2**·**6% during a 21 month community­based follow­up^18^ of 38 repaired women in Ethiopia. Even by 6 months’ follow­up, we recorded a higher proportion (5**·**6%) than noted among 233 women discharged with a closed fistula in Ethiopia (2**·**6%).^11^ The notable differences observed might be related to several factors, including the different follow­up periods, sample sizes, participant recruitment or diagnostic methods, fistula recurrence case definition, or the differences in sample characteristics across studies. Furthermore, most of the previously mentioned studies cited did not do a physical exam or dye test. More than half of the recurrences documented in our study occurred within the first 12 months following hospital discharge, with the maximum risk of recurrence within the first 6 months after discharge (37% of all recurrences). These findings indicate the need to identify and implement interventions that go beyond repair, which might be challenging given the barriers to engaging women after discharge, such as geographical distribution, transportation costs, and the absence of supportive priorities or resources in many fistula programmes.^29–31^ To our knowledge, although patients are often encouraged to return for a follow­up visit, most services provided by fistula treatment programmes are limited to hospital stay, including sexual and reproductive health counselling at discharge, psychological counselling, skill empowerment, literacy classes, or support groups before discharge.^14,15,20,32^ A rethink of fistula programming to include locally adapted follow­up mechanisms to prevent post­repair recurrence is needed to safeguard the health of women after fistula repair.^29^

More fistula recurrences were recorded in women with a damaged urethra or bladder neck and vaginal scarring at time of fistula surgery. Periurethral fistulas are more delicate and more likely to break down than are higher fistulas, and the role of vaginal scarring and status of bladder neck has already been described in the African context.^33,34^

Women reported that fistula recurrences happened during farming activities, walking, or sexual intercourse, confirming what has already been reported.^11–13,35^ However, the association between absence of sexual activity after repair and fistula recurrence should be interpreted with caution. First, the information was collected at the time of follow­up and therefore the directionality of the association cannot be established, and this characteristic might have changed because of fistula recurrence or residual incontinence (reverse causality). Second, divorce or abandonment might lead to socioeconomic precariousness, resulting in differential risk for recurrence. Third, some women were simply unable to have intercourse after fistula repair or only with great difficulty because of vaginal scarring or vaginal stenosis. Whatever the explanation, the findings contrast with the existing literature identifying sexual intercourse as a potential causative factor for fistula recurrence^11,13,23,35^ and warrant further research.^36^

More than a fifth of sexually active women of reproductive age in our study became pregnant at least once during the study follow­up. The observed pregnancy incidence was lower than what would be expected from women who have not experienced fistula. The low pregnancy incidence observed in our study might be related to infrequent sexual activity structuring differential risk of pregnancy during the follow­up period due to fear of fistula recurrence, lack of partner following hospital discharge, gynatresia, intrauterine scarring, upper urinary tract infection, or biological and physiological dysfunctions reported to be frequent after fistula surgery, such as amenorrhoea.^10,37,38^ Wilson and colleagues^18^ reported that women repaired for genital fistula frequently complained of infertility, which might be the explanation behind our findings. Furthermore, a study^39^ done in the African context has reported decreased fertility in women following a caesarean delivery, particularly in those undergoing emergency caesarean sections.

Most pregnancies occurred between 3 months and 24 months after discharge. Early pregnancies and their associated adverse neonatal outcomes observed in this study suggest that either childbearing desire is high among women after surgery or women are not empowered enough to make decisions about the timing of pregnancy, specifically regarding planning for delivery. At many repair hospitals, providers spend a lot of time counselling patients and people accompanying them that they will need a scheduled caesarean section and delivery in a hospital, and to use family planning methods to delay pregnancy after repair. However, this outcome is very challenging for providers and women to achieve.^40^ Therefore, a need exists to ensure that women and their partners are well informed of the need for elective caesarean section, given that caesarean section and obstetric care are free of charge in Guinea. Furthermore, current and future fistula programmes should include locally suitable post­discharge follow­up and management mechanisms for these women.

At first pregnancy after repair, we observed high rates of adverse maternal and neonatal outcomes (fistula recurrence, stillbirth, and maternal death), which are consistent with other reports from different African contexts, albeit with small sample sizes.^10,23,41^ In an 18­month longitudinal study in Niger and Mali,^41^ post­repair pregnancy­related adverse outcomes (two stillbirths and one suspicion of fistula recurrence) were recorded only in women who delivered without medical assistance. Furthermore, a review^23^ showed that after fistula surgery, women who delivered vaginally or by emergency caesarean section were at greatest risk of having adverse maternal and child health outcomes. In this study, all delivery­related fistula recurrences and stillbirths occurred in women who had vaginal delivery or emergency caesarean section. That women who already developed and lived with genital fistula had subsequent high incidences of stillbirth and fistula recurrence in a following pregnancy is very concerning. Loss of a child during the delivery associated with fistula is a traumatic experience;^16,17^ a repeated infant loss after repair is even more of a human and public health tragedy.^9,17^ Our findings show the need for interventions that will prevent occurrence of female genital fistula in women of childbearing potential and improve the health of those who receive treatment.

Our study has several limitations. First, follow­up time was short for some women who had undergone a fistula repair in 2015 and part of the sample was included retrospectively. Second, we did not identify cause(s) of urinary incontinence in women with residual incontinence.^11,42^ Third, the circumstances of fistula recurrence relied on women self­reporting the date of onset of severe urinary or faecal incontinence symptoms and preceding activity. Fourth, we did not use any fistula classification system to stratify by type of fistula in the analysis. Finally, because more women were living in urban areas at follow­up than at time of surgery, it is possible that they were at lower risk of having fistula recurrence, which would have underestimated the incidence rate.

Despite these limitations, this is the first study from Guinea to report on the recurrence of fistula, pregnancy, and childbirth after repair of female genital fistula with a sufficient sample size and a relatively long follow­up. This study adds to the existing body of knowledge on the topic and supports the feasibility of community follow­up in our context.^10,11^

Recurrence of female genital fistula seemed to be more frequent in Guinea than noted in previous reports from other sub­Saharan African countries. Women who undergo fistula surgery are still at risk of having adverse maternal and child health outcomes in Guinea. This risk underscores the need to rapidly identify locally suitable interventions to safeguard the health of these women so that, at a minimum, they do not develop a second fistula or lose their babies when they become pregnant after repair.

## Supplementary Material

Supplementary Appendix

## Figures and Tables

**Table 1:** Demographic and clinical characteristics at time of fistula surgery among eligible female study participants and eligible female non-participants, 2012–16 in Guinea

	Non-participants (n=201)	Study participants (n=481)	p value
Mean age at surgery, years (SD)	36·3 (12·6)	34·4 (12·4)	0·077
Residence	··	··	0·089
Available data	200 (>99%)	479 (>99%)	··
Rural	180 (90%)	449 (94%)	··
Urban	20 (10%)	30 (6%)	··
Mean duration of fistula symptoms, months (SD)	119·1 (11·7)	112·5 (11·6)	0·518
Marital status at surgery	··	··	0·838
Available data	195 (97%)	472 (98%)	··
Married or union	146 (75%)	339 (72%)	··
Other*	49 (25%)	133 (28%)	··
Occupation at surgery	··	··	0·922
Available data	198 (99%)	474 (99%)	··
Housewife	187 (94%)	445 (94%)	··
Other^†^	11 (6%)	29 (6%)	··
Level of education at surgery	··	··	0·769
Available data	192 (96%)	471 (98%)	
None	179 (93%)	442 (94%)	
Primary or higher	13 (7%)	29 (6%)	
Mean parity (SD)	3·6 (2·7)	3·6 (2·5)	0·857
Location of delivery	··	··	0·183
Available data	200 (>99%)	478 (99%)	··
Home	69 (35%)	191 (40%)	··
Health structure	131 (66%)	287 (60%)	··
Method of delivery	··	··	0·555
Available data	201 (100%)	479 (>99%)	··
Vaginal	127 (63%)	314 (66%)	··
Caesarean section	74 (37%)	165 (34%)	··
Neonatal outcome	··	··	0·027
Available data	196 (98%)	471 (98%)	··
Alive	24 (12%)	33 (7%)	··
Stillborn	172 (88%)	438 (93%)	··
Type of obstetric fistula	··	··	0·063
Available data	201 (100%)	480 (>99%)	··
Vesicovaginal fistula	184 (92%)	457 (95%)	··
Other^‡^	17 (8%)	23 (5%)	··
Number of previous repairs	··	··	0·105
Available data	192 (96%)	479 (>99%)	··
None	102 (53%)	298 (62%)	··
One or more	90 (47%)	181 (38%)	··
Urethral involvement	··	··	0·916
Available data	181 (90%)	465 (97%)	··
No	105 (58%)	274 (59%)	··
Yes	76 (42%)	191 (41%)	··
Status of bladder neck	··	··	0·873
Available data	187 (93%)	462 (96%)	··
Normal	109 (58%)	266 (58%)	··
Damaged	78 (42%)	196 (42%)	··
Vaginal scarring	··	··	0·521
Available data	168 (84%)	439 (91%)	··
No	74 (44%)	177 (40%)	··
Yes	94 (56%)	262 (60%)	··
Route of repair	··	··	0·663
Available data	200 (>99%)	481 (100%)	··
Vaginal	195 (98%)	466 (97%)	··
Abdominal	5 (3%)	15 (3%)	··
Continence status at discharge	··	··	0·006
Available data	196 (98%)	481 (100%)	··
Closed and continent	169 (86%)	447 (93%)	··
Closed and not continent	27 (14%)	34 (7%)	··

* Single, widowed, divorced, or separated.

† Office worker, farming, market vendor, or student.

‡ Rectovaginal fistula or both vesicovaginal fistula and rectovaginal fistula.

**Table 2:** Selected demographic characteristics of study participants at surgery and follow-up

	In hospital at surgery (n=481)	At follow-up visit (n=481)	p value
Residence	··	··	<0·0001
Rural	449 (93%)	419 (87%)	··
Urban	30 (6%)	62 (13%)	··
Unknown	2 (<1%)	0	··
Marital status	··	··	0·370
Married or union	339 (70%)	360 (75%)	··
Other*	133 (28%)	121 (25%)	··
Unknown	9 (2%)	0	··
Occupation	··	··	<0·0001
Housewife	445 (93%)	311 (65%)	··
Other occupation^†^	29 (6%)	170 (35%)	··
Unknown	7 (1%)	0	··

* Single, widowed, divorced, or separated.

† Office worker, farming, market vendor, or student.

**Table 3:** Incidence of fistula recurrence, residual urinary incontinence, and pregnancy

	Fistula recurrence post-repair		Residual urinary incontinence		First pregnancy after repair	
	Events	Incidence (95% CI)	Events	Incidence (95% CI)	Events	Incidence (95% CI)
**Cumulative incidence per 1000 person-years**						
Total	73	71·0 (56·5–89·3)	24	23·1 (14·0–36·2)	67	106·0 (83·2–134·3)
**Cumulative incidence by 6 month study period**						
6 months	27	5·6% (3·9–8·1)	4	0·8% (0·3–2·2)	5	1·7% (0·7–3·9)
12 months	12	8·2% (6·1–11·1)	4	1·7% (0·9–3·4)	19	8·1% (5·5–11·8)
18 months	17	12·4% (9·7–15·9)	7	3·4% (2·1–5·6)	24	17·6% (13·5–22·7)
24 months	10	15·5% (12·3–19·4)	1	3.7% (2·3–6·0)	9	21·9% (17·3–27·6)
30 months	4	16·9% (13·5–21·0)	1	4·1% (2·5–6·5)	6	25·2% (20·2–31·2)
≥36 months	3	18·4% (14·8–22·8)	7	10·3% (5·2–19·6)	4	28·4% (22·8–35·0)

**Table 4:** Cumulative incidence of study outcomes for selected study variables among women discharged with a closed fistula, 2012–16 in Guinea

	Cumulaive incidence	Cumulative incidence per 1000 person-years (95% CI)	Rate ratio	p value
**Fistula recurrence**				
Pregnancy status	··	··	1·2 (0·8–1·7)	0·3061
No	63	73·0 (57·0–93·4)	··	··
Yes	10	60·7 (32·7–112·9)	··	··
Sexual activity				
No	43	142·6 (105·7–192·2)	3·4 (2·1–5·7)	<0·0001
Yes	30	41·3 (28·9–59·1)	··	··
Urethral involvement	··	··	2·7 (1·6–4·6)	<0·0001
No	25	42·2 (28·5–62·5)	··	··
Yes	45	113·8 (85·0–152·5)	··	··
Status of bladder neck	··	··	1·9 (1·2–3·2)	0·0032
Normal	29	51·1 (35·5–73·5)	··	··
Damaged	41	98·7 (72·7–134·0)	··	··
Vaginal scarring	··	··	1·7 (1·0–3·0)	0·0291
No	19	49·9 (31·8–78·2)	··	··
Yes	47	82·7 (62·1–110·1)	··	··
**Residual incontinence**				
Pregnancy status	··	··	1·9 (0·6–4·9)	0·1011
No	18	18·8 (11·9–29·9)	··	··
Yes	6	35·2 (15·8–78·5)	··	··
Sexual activity	··	··	1·2 (0·9–1·5)	0·3557
No	7	18·8 (9·0–39·5)	··	··
Yes	17	22·5 (14·0–36·2)	··	··
Urethral involvement	··	··	1·6 (0·6–3·8)	0·1431
No	11	17·8 (9·9–32·2)	··	··
Yes	13	27·8 (16·1–47·9)	··	··
Status of bladder neck	··	··	1·8 (0·7–4·5)	0·0847
Normal	10	16·5 (8·9–30·8)	··	··
Damaged	14	29·4 (17·4–49·6)	··	··
Vaginal scarring	··	··	1·6 (0·6–4·5)	0·1612
No	7	17·2 (8·2–36·1)	··	··
Yes	17	27·0 (16·8–43·5)	··	··
**Pregnancy**				
Urethral involvement	··	··	1·3 (0·8–2·1)	0·1603
No	36	95·1 (68·6–131·8)	··	··
Yes	29	122·0 (84·8–175·6)	··	··
Status of bladder neck	··	··	1·0 (0·9–1·1)	0·4668
Normal	38	102·4 (74·5–140·7)	··	··
Damaged	24	99·9 (67·0–149·1)	··	··
Vaginal scarring	··	··	1·0 (0·6–1·8)	0·4256
No	24	99·2 (66·5–148·1)	··	··
Yes	37	104·5 (75·7–144·2)	··	··
Fistula status at discharge	··	··	2·2 (0·6–5·9)	0·0798
Closed and dry	63	102·3 (79·9–130·9)	··	··
Closed with residual incontinence	4	224·7 (84·3–598·7)	··	··

Some variables are missing data as these could not be collected from certain women.

## Research in context

### Evidence before this study

Recurrence of fistula and pregnancy after repair of female genital fistula is not well documented in Guinea and sub-Saharan Africa. We searched PubMed for articles published between Jan 1, 1970, and March 31, 2017, with no language restrictions using the terms “post-repair”, “fistula”, “leakage”, “recurrence”, “pregnancy”, “delivery”, “childbirth”, “birth”, and “reintegration”. We found two recent reviews and two additional original studies reporting on fistula recurrence and pregnancy after repair of female genital fistula. The major findings were that most women who become pregnant after fistula surgery deliver either by emergency caesarean section or vaginally, which increases the risk of adverse maternal and neonatal outcomes, including stillbirths, recurrence of the fistula, or maternal death. However, studies do not provide a clear estimate of post-repair fistula recurrence and pregnancy rates. Additionally, most studies used small samples, were done at hospitals, and had short follow-up time.

### Added value of this study

Our study fills a gap in knowledge about the health of women after fistula surgery. As far as we know, this study is the first of its kind from Guinea to report on the recurrence of fistula, pregnancy, and childbirth after repair of female genital fistula, with a sufficient sample size recruited across the country and a relatively long follow-up time. The study adds to the existing body of knowledge on this topic and supports the feasibility of community follow-up in our context. The results provide evidence to guide the design and implementation of interventions that target post-repair reintegration.

### Implications of all the available evidence

Combining evidence from this study with existing evidence suggests that women who undergo female genital fistula surgery in Guinea are still at high risk of fistula recurrence and adverse maternal and neonatal outcomes during their reintegration process. Overall, recurrence of female genital fistula was more frequent in Guinea than noted in previous reports from other sub-Saharan African countries. Pregnancy occurrence was relatively low compared with what would be expected and adverse maternal and neonatal outcomes were very common, particularly among women who delivered vaginally or by emergency caesarean section. Our findings underscore the need to rapidly identify locally suitable interventions to safeguard the health of women and that of their babies when they become pregnant after repair.
