# Supplementary material for: Fistula recurrence, pregnancy, and childbirth following successful closure of female genital fistula in Guinea: a longitudinal study
Source: Lancet Glob Health. Author manuscript; Available in PMC 2019 May 22. (PMC6530985; doi:10.1016/S2214-109X(17)30366-2)
Supplement: Supplementary Appendix [file NIHMS1018216-supplement-Supplementary_Appendix.pdf]

THE LANCET
Global Health

Supplementary appendix

This appendix formed part of the original submission and has been peer reviewed. We post it as supplied by the authors.

Supplement to: Delamou A, Delvaux T, El Ayadi AM, et al. Fistula recurrence, pregnancy, and childbirth following successful closure of female genital fistula in Guinea: a longitudinal study. *Lancet Glob Health* 2017; published online Sept 20. http://dx.doi.org/10.1016/S2214-109X(17)30366-2.

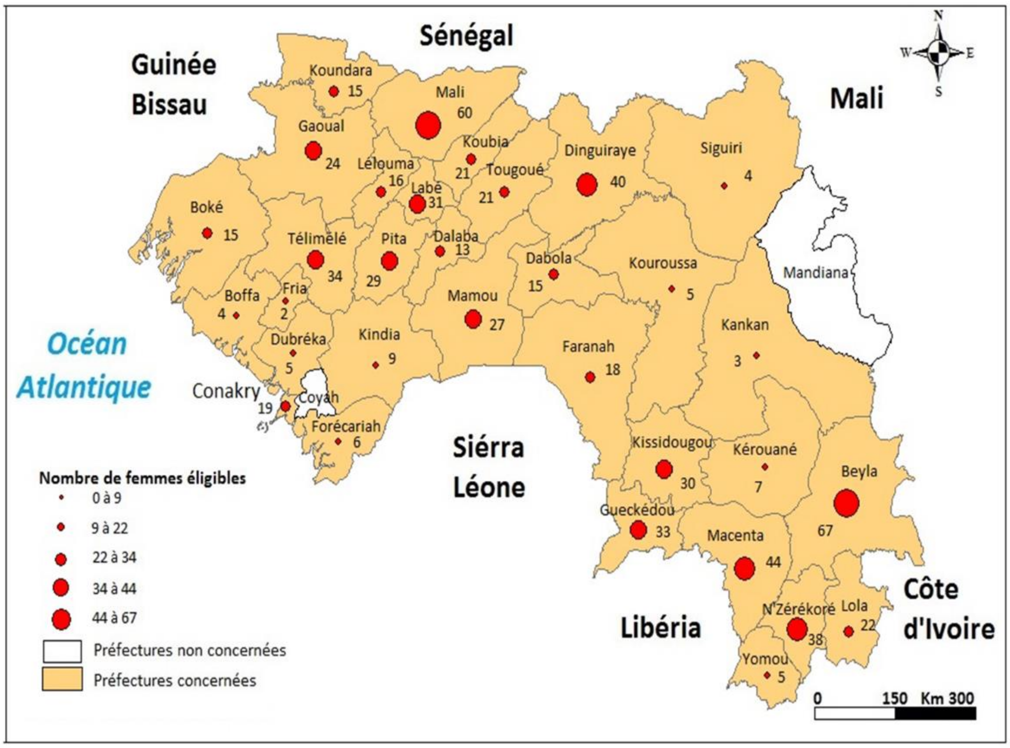

Figure (a)

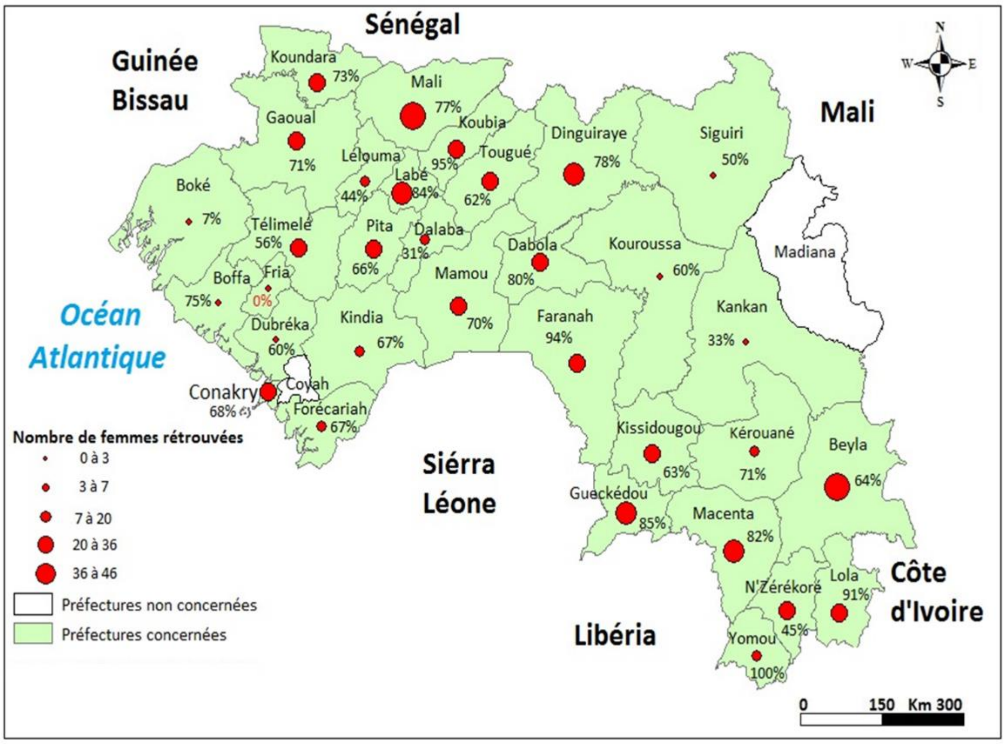

Figure (b)

Additional file 1. Distribution across the country of eligible women (figure a; n=682) and those women included in the study (figure b; n=481), 2012 to 2016, Guinea.
